# The Use of Open-Source Online Course Content for Training in Public Health Emergencies: Mixed Methods Case Study of a COVID-19 Course Series for Health Professionals

**DOI:** 10.2196/42412

**Published:** 2023-02-23

**Authors:** Nadine Ann Skinner, Nophiwe Job, Julie Krause, Ariel Frankel, Victoria Ward, Jamie Sewan Johnston

**Affiliations:** 1 Stanford Center for Health Education Stanford University Stanford, CA United States; 2 Stanford Center for Health Education Stanford University Cape Town South Africa; 3 Last Mile Health Boston, MA United States; 4 TechChange Washington, DC United States; 5 Pediatrics Stanford University School of Medicine Stanford, CA United States

**Keywords:** global health education, digital education, digital health, COVID-19 pandemic, health care access, partnerships for health, community health workers, remote learning

## Abstract

**Background:**

The onset of the COVID-19 pandemic generated an urgent need for credible and actionable information to guide public health responses. The massive open-source online course (MOOC) format may be a valuable path for disseminating timely and widely accessible training for health professionals during public health crises; however, the reach and effectiveness of health worker–directed online courses during the pandemic remain largely unexplored.

**Objective:**

This study investigated the use of an open-source online course series designed to provide critical COVID-19 knowledge to frontline health workers and public health professionals globally. The study investigated how open-source online educational content can be optimized to support knowledge sharing among health professionals in public health emergencies, particularly in resource-limited contexts.

**Methods:**

The study examined global course enrollment patterns (N=2185) and performed in-depth interviews with a purposive subsample of health professionals enrolled in the course series (N=12) to investigate the sharing of online content in pandemic responses. Interviewed learners were from Ethiopia, India, Kenya, Liberia, Malawi, Rwanda, Thailand, Uganda, the United Arab Emirates, and the United States. Inductive analysis and constant comparative methods were used to systematically code data and identify key themes emerging from interview data.

**Results:**

The analysis revealed that the online course content helped fill a critical gap in trustworthy COVID-19 information for pandemic responses and was shared through health worker professional and personal networks. Enrollment patterns and qualitative data illustrate how health professionals shared information within their professional networks. While learners shared the knowledge they gained from the course, they expressed a need for contextualized information to more effectively educate others in their networks and in their communities. Due to technological and logistical barriers, participants did not attempt to adapt the content to share with others.

**Conclusions:**

This study illustrates that health professional networks can facilitate the sharing of online open-source health education content; however, to fully leverage potential benefits, additional support is required to facilitate the adaptation of course content to more effectively reach communities globally.

## Introduction

### Background

As the COVID-19 pandemic began to spread rapidly in 2020, the global health community needed to act quickly to curb the spread of the highly infectious virus. To understand how to prevent and treat COVID-19 infections, communities worldwide turned to local health professionals for answers, creating an urgent need for trustworthy information to guide public health responses and to inform health care workers and the general public [1]. The rapidity of the response required for the public health emergency meant that health professionals had to rely on the limited training available. This study examined global enrollment in an open-source, self-paced, online library of COVID-19 courses developed in response to the pandemic. It aimed to better understand how the massive open-source online course (MOOC) format can be used to rapidly educate and support the sharing of knowledge among health professionals during health crises. In particular, the study aimed to understand how a single MOOC was taken up by learners across vastly different contexts and countries with differing languages, cultural backgrounds, pandemic experiences, technology access, and public health systems.

Given the novelty of the SARS-CoV-2 virus, as well as the new roles required to respond to and manage the pandemic, frontline health professionals and community health workers were in need of specific training and support [2-5]. Research has shown that frontline health care staff training in skills and knowledge related to disease outbreaks can lead to improvements in awareness of the disease, screening and reporting, and more proactive involvement in disease prevention and control efforts, as well as increased confidence to cope with managing disease outbreaks [4,6]. However, community health workers and other frontline health professionals, especially in resource-limited settings, are not often well supported and equipped with the training and resources needed to contain the spread of pandemics, such as COVID-19, despite the pivotal role that they play in the response [2-4,7]. While there is a wide variation in how community health workers and frontline health workers are defined and trained, and what tasks they are expected to carry out, in the midst of the disease outbreak, their roles expanded from the provision of ongoing public health services to also include pandemic mitigation measures, such as case detection, contact tracing, and triaging patients for care, amidst overwhelmed health systems [2,4].

Digital health content and tools have been used successfully to support the training and education of frontline and community-based health care workers [5,6,8-11]. With the requirement for social distancing, as well as countrywide and regional lockdowns caused by the COVID-19 pandemic, reliance on a variety of digital education tools has grown [10]. The demand for information on the novel coronavirus, particularly at the start of the pandemic, was strong, as evidenced by substantial enrollment in COVID-19–focused online courses globally [12-14]. Yet, there is still a gap in the literature regarding the specific ways in which health professionals used online course content in their pandemic responses globally and the ways in which health care professionals can be better supported to use and share knowledge from online content in their health emergency responses [15].

### Intervention

In April 2020, a consortium of 7 international organizations with expertise in health education, community health program implementation, global innovation, and digital technologies convened to develop a novel curricula and platform for community-based health workers and public health professionals responding to COVID-19 outbreaks in their communities. The consortium developed the COVID-19 Digital Classroom as a library of free, open-source, and mobile-friendly online courses. The series of resources consisted of 8 self-paced online courses on different topics related to COVID-19, including general information about the virus, prevention and protection, contact tracing, home-based care and isolation, community-based surveillance, risk communication and community engagement, mental health and wellness, and continuity of primary health care during COVID-19. Each course was estimated to take between 45 and 60 minutes to complete, with the exception of 1 course on contact tracing that was estimated to take over 90 minutes to complete.

The consortium designed the COVID-19 Digital Classroom specifically to support community-based health workers in low- and middle-income countries (LMICs). The courses were first developed in English (and later translated into additional languages) and included a variety of interactive activities, video animations, and infographics to overcome literacy limitations and language barriers. The video content animations were designed as standalone pieces of content that could be shared on social media or downloaded and shared via other channels, such as WhatsApp, to address limitations in bandwidth or technology access. The first course was launched in English in June 2020, with subsequent English-language courses added to the series through December 2020. The series was promoted to health workers and the general public by members of the consortium through webinars, emails, and social media channels.

### Objectives

This study examined the use of this MOOC series in order to better understand how this type of digital education content can be optimized to reach and support knowledge sharing among community-based health professionals in public health emergencies, particularly in resource-limited contexts. We examined global enrollment patterns to explore demand, ability to access online content, and sharing of course content among learners. Through in-depth interviews, we investigated the ways in which course content was used, adapted, and shared among health professionals as part of the COVID-19 pandemic response.

## Methods

### Data Sources

The study leverages the following 2 sources of data: (1) course enrollment data between June 2020 and July 2021 (N=2185) and (2) in-depth interviews with a purposive sample of health professionals, with a focus on enrollees who shared course content as part of community-based pandemic responses (N=12).

#### Enrollment Data

Descriptive enrollment data provided a framework for understanding demand and ability to access the online courses. These data also served as the sampling frame for constructing a purposive qualitative sample. We examined data collected through a registration survey administered to all enrollees of the course series, including information on learners’ country of residence, gender, institutional affiliation, profession, and type of involvement in the COVID-19 response, and how learners heard about the course series. We focused on the first year of enrollment in the English-language version of the course series that was first launched between June 2020 and July 2021 to better understand how content was used in the early pandemic response.

#### Interview Data

For in-depth interviews, the research team selected a purposive sample of learners (N=12) across health professions from different regions globally. Because the course series was designed for community-based health workers, sampling focused on identifying health professionals with experience using and sharing course content as part of community-based pandemic responses.

The study considered the following learners for recruitment: (1) learners who indicated that they had shared course content with others in their network in a voluntary follow-up course satisfaction survey (administered by the consortium in December 2020; N=112); (2) learners in a community-based health worker role or in a supervisory role in a position to share information with community-based health workers (ie, doctors, nurses, health worker trainers/supervisors, and technical assistance providers) and at an organization with more than one enrollee in the course series; and (3) learners holding an educator role at a higher education institution with more than one enrollee in the course series. Learners who indicated that they did not consent to be contacted further in the follow-up course satisfaction survey were excluded from recruitment.

A total of 119 learners met the purposive sampling criteria and were recruited to participate via email in the study. Learners were sent an introductory recruitment email, and those who did not respond to the initial email were sent several follow-up email requests. Fourteen learners responded with willingness to participate in an in-depth interview (11.8% response rate). The research team was able to schedule in-depth interviews with a sample of 12 of these learners and made efforts to ensure representation across geographic regions and from LMICs. No additional recruitment was deemed necessary as the research team determined saturation was achieved.

As illustrated in Table 1, the 12 interview participants represented a diversity of geographic regions, with 42% (5/12) from Sub-Saharan Africa, followed by 25% (3/12) from North America and 25% (3/12) from South/Southeast Asia. Half (6/12, 50%) of the interviewed learners identified as female. The majority of interviewed learners (7/12, 58%) indicated affiliation with nongovernmental organizations (NGOs). The remaining interviewed learners held roles in governments (2/12, 17%), academic institutions (2/12, 17%), or intergovernmental organizations (1/12, 8%). Interviewed learners were doctors (3/12, 25%), health worker trainers or supervisors (3/12, 25%), community-based health workers (2/12, 17%), technical assistance providers (2/12, 17%), or educators (2/12, 17%). All were involved in community-based COVID-19 response activities, with nearly all (10/12, 83%) involved in risk communication and community engagement.

The 12 in-depth interviews were conducted one-on-one in English via videoconference by 2 investigators (NAS and NJ) using a semistructured interview guide. The interviewers asked learners about their experiences with the curriculum and their roles in using, adapting, and disseminating the curriculum. The interviews were audio recorded and transcribed. Interviews lasted between 20 and 57 minutes, with a mean duration of 38 minutes.

**Table 1:** In-depth interview sample characteristics (N=12).

Characteristic			Value, n (%)
**Country group**			
	High income	4 (33)	
	Upper-middle income	1 (8)	
	Lower-middle income	4 (33)	
	Low income	3 (25)	
**Region**			
	Middle East	1 (8)	
	North America	3 (25)	
	South/Southeast Asia	3 (25)	
	Sub-Saharan Africa	5 (42)	
**Gender**			
	Female	6 (50)	
	Male	6 (50)	
**Institutional affiliation**			
	Academic institution	2 (17)	
	Government	2 (17)	
	Nongovernmental organization	7 (58)	
	Private sector	1 (8)	
**Profession**			
	Community health worker	2 (17)	
	Doctor	3 (25)	
	Educator	2 (17)	
	Health worker trainer/supervisor	3 (25)	
	Technical assistance provider	2 (17)	
**COVID-19 response involvement^a^**			
	Contact tracing	3 (25)	
	Risk communication and community engagement	10 (83)	
	Surveillance	4 (33)	
	Testing	1 (8)	
	Treatment	2 (17)	
	Other	2 (17)	
	None	0 (0)	

^a^Participants were involved in multiple types of COVID-19 responses, and hence, percentages do not add to 100.

### Data Analysis

Enrollment data were summarized using descriptive statistics (response rate, mean, and SD). Statistical analyses were performed using Stata SE version 15 (StataCorp). The interview transcripts were analyzed through thematic coding using Dedoose (SocioCultural Research Consultants). Inductive analysis and constant comparative methods were used to systematically code data and identify key themes emerging from interview data. Each interview transcript was independently coded by a member of the team (NAS, NJ, and JSJ) and then independently reviewed by a second coder. None of the transcripts were coded by investigators who conducted the interview. The research team met multiple times to confer and calibrate the coding interpretation and to further refine and recalibrate coding schemes. The analysis was concluded with a final pass of the transcripts by 2 coders.

### Ethics Approval

Informed consent was obtained from all interview participants. Approval for all aspects of this study, including for consent, outreach, data collection, interviewing, and data analysis, was obtained from the Stanford University School of Medicine Institutional Review Board (protocol number: 61266).

## Results

### Enrollment Patterns

To investigate the types of learners seeking out open-source online education in the first year of the COVID-19 pandemic, we examined the characteristics of the 2185 learners who enrolled in at least one of the courses in the course series between June 2020 and July 2021. As shown in Table 2, while enrollees were distributed globally across 104 countries, a majority of learners were from North America (1551/2185, 71.0%) and primarily the United States (70.3%), followed by Sub-Saharan Africa (315/2185, 14.4%). Among all learners, 12.2% (266/2185) were from lower-middle–income countries and 5.4% (118/2185) were from low-income countries, as classified by the World Bank [16]. The preferred language across learners was predominantly English (1664/2185, 76.2%), which was not unexpected given the focus on enrollment in the English-language version of the course series, which was initially the only version available and the only one examined in this data set. Nevertheless, 3.8% (83/2185) of all enrollees and 7.8% (30/384) of enrollees in LMICs indicated preference for a language other than English.

Learners enrolled in the course series were predominantly affiliated with academic or research institutions, including health worker training institutions, for example, nursing schools (686/2185, 31.4%); NGOs and civil society organizations (535/2185, 24.5%); clinical settings (ie, hospitals, health facilities, and clinics; 331/2185, 15.2%); or governments (316/2185, 14.5%). However, in LMICs, 50.0% (192/384) of learners were affiliated with NGOs or civil society organizations. Among all learners, students made up 25.5% (558/2185) of learners, but nearly all were located in high-income or upper-middle–income countries. Over a third of all learners (762/2185, 34.9%) were frontline health providers (ie, clinical officers, community-based health workers, doctors, or nurses/midwives). Over 70% of all learners were involved in some sort of COVID-19 response, while nearly 90% of learners in LMICs were involved with COVID-19 response.

As shown in Table 3, enrollment patterns indicated that learners heard about the course series through personal and professional networks. Overall, 30.1% (658/2185) of learners heard about the MOOC from their employers, while another 8.5% (186/2185) heard about the MOOC from friends or colleagues. The proportion hearing about the series from friends or colleagues was higher in LMICs at 21.6% (83/384). In LMICs, the proportion hearing about the MOOC from direct promotion by the consortium that developed the course series was also higher at 44.3% (170/384).

At registration, learners were given a chance to identify their specific organization affiliation, and 1504 learners identified as being affiliated with 779 unique organizations, of which 110 organizations had more than two learners. In LMICs, 303 learners identified as being affiliated with 221 unique organizations, of which 35 organizations had more than two learners. The average number of learners per organization with multiple learners was lower in LMICs (4.9 learners) than in the overall sample (7.6 learners). This analysis included only learners identified through enrollment registration analytics.

**Table 2:** Global learner characteristics.

Characteristic				Overall (N=2185), n (%)		LMICs^a^ (N=384), n (%)
**Country group**						
	High income			1631 (74.7)		N/A^b^
	Upper-middle income			165 (7.6)		N/A
	Lower-middle income			266 (12.2)		N/A
	Low income			118 (5.4)		N/A
	Not specified			5 (0.2)		N/A
**Region**						
	North America			1551 (71.0)		22 (5.7)
	Sub-Saharan Africa			315 (14.4)		237 (61.7)
	Europe & Central Asia			75 (3.4)		4 (1.0)
	South Asia			75 (3.4)		75 (19.5)
	Latin America & Caribbean			67 (3.0)		9 (2.3)
	East Asia & Pacific			56 (2.6)		37 (9.6)
	Middle East & North Africa			41 (1.9)		22 (5.7)
	Not specified			5 (0.2)		0 (0.0)
**Preferred language**						
	English			1664 (76.2)		298 (77.6)
	Another language^c^			83 (3.8)		30 (7.8)
	Not specified			483 (20.0)		56 (14.6)
**Gender**						
	Female			1531 (70.1)		154 (40.1)
	Male			505 (23.1)		213 (55.5)
	Nonbinary			13 (0.6)		3 (0.8)
	Not specified			136 (6.2)		14 (3.7)
**Institutional affiliation**						
	Academic/research institution			686 (31.4)		54 (14.1)
	Government			316 (14.5)		48 (12.5)
	Hospital, health facility, or clinic			331 (15.2)		31 (8.1)
	Intergovernmental/donor agency			55 (2.5)		22 (5.7)
	Nongovernmental organization/civil society			535 (24.5)		192 (50.0)
	Private sector			107 (4.9)		11 (2.9)
	Self-employed/not employed			155 (7.1)		26 (6.8)
**Profession**						
	Educator			61 (2.8)		15 (3.9)
	**Frontline health worker**					
		Clinical officer	23 (1.0)		10 (2.6)	
		Community-based health worker	379 (17.4)		34 (8.9)	
		Doctor	92 (4.2)		45 (11.7)	
		Nurse midwife	268 (12.3)		21 (5.5)	
	Government official			37 (1.7)		8 (2.1)
	Health educator			78 (3.6)		13 (3.4)
	Health worker trainer/supervisor			84 (3.8)		23 (6.0)
	Program manager			222 (10.2)		87 (22.7)
	Student			558 (25.5)		19 (5.0)
	Technical assistance provider			114 (5.2)		45 (11.7)
	Other health professional^d^			90 (4.1)		21 (5.5)
	Other professional^e^			222 (10.2)		43 (11.2)
**COVID-19 response involvement^f^**						
	Contact tracing			485 (22.2)		79 (20.6)
	Risk communication and community engagement			893 (40.9)		278 (72.4)
	Surveillance			303 (13.9)		111 (28.9)
	Testing			169 (7.8)		36 (9.4)
	Treatment			333 (15.3)		48 (12.5)
	Other			511 (23.4)		123 (32.0)
	None			639 (29.2)		40 (10.4)

^a^LMICs: low- and middle-income countries.

^b^N/A: not applicable.

^c^Other languages preferred (in order of the highest to lowest demand) were Spanish, French, Portuguese, Arabic, Hindi, Bengali, Burmese, Indonesian, Russian, German, Italian, Swahili, Ukrainian, Khmer, Krio, and Telugu.

^d^Other health professionals included dentists, environmental health and safety professionals, epidemiologists, medical assistants, nutritionists, pharmacists, psychologists, social workers, and case managers.

^e^Other professionals included human resource professionals, librarians, media specialists, researchers, translators, and other unspecified professions.

^f^Enrollees could select multiple types of COVID-19 response involvements, and hence, percentages do not add to 100.

**Table 3:** Learner networks.

Variable			Overall (N=2185)		LMICs^a^ (N=384)
**How did you learn about the course series?^b^, n (%)**					
	Consortium promotion	378 (17.3)		170 (44.3)	
	Friend/colleague recommendation	186 (8.5)		83 (21.6)	
	Employer recommendation	658 (30.1)		78 (20.3)	
	Internet search	152 (7.0)		44 (11.5)	
	School requirement/recommendation	408 (18.7)		1 (0.3)	
	Social media	91 (4.2)		47 (12.2)	
	Other	347 (15.9)		23 (6.0)	
**Learners identifying organizational affiliation, n (%)**			1,504 (68.8)		303 (78.9)
	Learners at organizations with no other learners	669 (44.5)		186 (61.4)	
	Learners at organizations with 2-5 learners	238 (15.8)		53 (17.5)	
	Learners at organizations with 6-10 learners	106 (7.1)		35 (11.6)	
	Learners at organizations with 11-30 learners	143 (9.5)		29 (9.6)	
	Learners at organizations with >30 learners	348 (23.1)		0 (0.0)	
Unique organizations identified by learners, n			779		221
Organizations with two or more learners, n			110		35
Learners per organization among organizations with two or more learners, mean (SD)			7.6 (14.4)		4.9 (4.1)

^a^LMICs: low- and middle-income countries.

^b^Enrollees could select multiple ways of learning about the course series, and hence, percentages do not add to 100.

### In-Depth Interviews

Three major themes emerged in the thematic analysis. First, the COVID-19 Digital Classroom helped fill a critical gap in trustworthy COVID-19 training information globally, motivating learners to share the course series within their personal and professional networks. Second, although the comprehensive nature of the MOOC provided valuable information, the density of the course series made it difficult to navigate and use with all audiences, especially frontline and community-based health workers, as well as with the general public. Third, while participants shared the knowledge they gained from the courses, the vast majority of participants did not attempt to adapt the courses (eg, make content changes, such as add translations into local languages or add local contextual information, or make technical changes, such as disseminate the courses via different modalities including SMS text messages) to share within their communities, despite their expressed need, due to technological and logistical barriers.

#### Theme 1

Interviews revealed that in the early stages of the pandemic, there was a major need for trustworthy information about COVID-19 alongside an expectation that health professionals fill those gaps within communities. One community health worker from Kenya stated:

We were really not having an idea what we [were] dealing with. The community was expecting us to give them so much information and at that particular time we didn't have it, especially because there was no information around about COVID.

#158

Respondents shared that they sought out information from a variety of online sources to rapidly access new knowledge to respond to the COVID-19 pandemic. In addition to the COVID-19 Digital Classroom course series, respondents sought information from the World Health Organization, regional health organizations, the Centers for Disease Control in various countries and regions, their countries’ ministries and departments of health, media sources, and higher education institutions, as illustrated in the following statement by a health professor in Ethiopia:

I believe that because when you give advice, you need to be updated with what is new and what is going on. I used to be informed about any updates [from] the Digital Classroom and the different sources, definitely the WHO website. I followed the news as well [for] continuous updates.

#119

Several of the interview participants reflected on their responsibility as health care professionals to be informed in order to support their community. One community health worker from the United States stated:

Everybody just didn't know what to do, and I just wanted to be able to position myself and be able to sign up for the courses so I can be well versed and well-rounded and know how to assist those that were in need during this pandemic.

#615

The personal and professional networks of the health care workers enabled access to the MOOC. Many of the respondents learned about the course series through recommendations from employers or colleagues. Sharing of the COVID-19 Digital Classroom itself (ie, recommending that others within the network enroll in the courses) occurred primarily through several informal channels, including online messages and conversations, or via social media. Interviewed learners stated that they recommended the courses by name, shared the link to the courses through their networks, and often enrolled in the courses based on recommendations. In discussing this process, a learner from Ethiopia shared:

Whenever someone found a piece or documents we would share and see how we could bring a resource to share in our setup… There are two of my colleagues that did it [completed the course] that I know of... because…. we keep on asking each other for the latest information, and so when I got the link I also forwarded it to several colleagues.

#782

Interviewees indicated that the course series filled a knowledge gap in basic information and built confidence in their understanding of the pandemic. The interviewed learners indicated that they incorporated the knowledge gained from the courses into policies and protocols for working with patients, and the courses served as ongoing resources for their pandemic response. A doctor from Kenya shared:

Because you're a doctor you're expected to understand something. You can’t tell your patients ‘I don't know what it is. It's a new disease.’ That's not right. So at least I had to be able to get some information on how people can protect themselves. In fact, out of this information I was able to learn a lot. It helped us develop a process for our organization, a process for people with NCDs who are, for example, at a higher risk, with precautions they need to take.

#891

The interviewed community health workers and frontline health workers also reported that they added the presented information into their community workshops, and the doctors, professors, and health worker trainers incorporated the information into their trainings and classes for their health care students and community health workers. One community health worker described how having the knowledge and resources built her confidence in being able to do her work in her community. In referring to a workshop she led in her community, she stated “I hate when I stand before people and do not have information… the video is very good” [#158]. This learner went on to share that the course content was also used as a tool for communication with her patients:

I even remember at one particular time I played one of the videos with the class members to shed light on our hand hygiene and skills that I actually picked from the lessons, so I was even more empowered than before, because I was able to describe [the process].

#158

#### Theme 2

The second theme that emerged from the interviews was that while the COVID-19 Digital Classroom course series and the knowledge sharing that occurred through the respondents’ networks enabled them to access critical COVID-19 information, the density of the course series made the consumption and sharing of content more challenging, especially with community health worker audiences and community members. In discussing the challenge of using the COVID-19 Digital Classroom course series with community health workers, a health professional from Malawi said:

With community health workers, we have two groups. We have clinicians and nurses, who will be able to understand the course. Then we have the assistants, who are connected to the community in remote areas and provide basic health care, like vaccines, bring [people] to the clinics, [and] family planning. Most clinicians and nurses, they can be able to understand this, the courses, but the assistants may need a little bit more assistance for them to understand the courses.

#782

Many respondents indicated that there remained a large unmet need for resources on COVID-19 that could be more easily shared and used within their local communities. The health care professionals interviewed indicated that they needed resources to communicate with the general public, as well as resources to support the learning of health care workers they needed to train. Respondents expressed a need for additional scaffolding support to help navigate the dense information offered in the COVID-19 Digital Classroom course series through thoughtful platform design. They indicated a need for content structuring and platform functionality that could better facilitate the sharing of recommendations and encourage instructor, learner, and peer interactions, which would also help navigate the learning process. A doctor in Rwanda stated:

I think it became really clear really quickly that if we can't get the protection, the knowledge, the tools to communities we're not going to fight this virus, and we also saw that pre-existing trust or mistrust in the health system, made a huge difference in whether or not people were able to quickly adapt and adopt the recommendations that were being sent out around the world. So, for us, we saw locally, our role changed.

#560

As they embraced the new responsibility to share COVID-19 information rapidly in their communities, respondents shared how the ability to adapt existing online content, including the COVID-19 Digital Classroom, and easily share key recommendations would allow for the incorporation of local contextualization that could make a difference in how information is perceived in communities.

#### Theme 3

The final theme to emerge from the qualitative analysis was that despite participants’ interest in having the course series adapted to meet their local needs, due to technological and logistical barriers, the vast majority of participants did not attempt large-scale content adaptations of the course series (eg, adding translations into local languages, adding local contextual information on COVID-19 responses, or adding local contextual cultural information). Only 1 large international organization, who partnered directly with the consortium, led full technological adaptation of the curriculum into an SMS text message format to expand access to course content. The international organization partnered with the consortium and a higher education institution to provide the course materials to those organizations who partnered with their LMIC offices and who only had access to basic phones.

Interviewed learners indicated they wanted adaptations that would involve adding, removing, and editing components to make the content relevant to their local context. They also expressed the need for translations of course materials in local languages. For example, a doctor in Rwanda desired an adapted version with key points, and this doctor stated “…the key points, and if you want to dive in deeper, here's the way to do it, but otherwise here [are] the key takeaways you need to know to make educated decisions for your health and your patients.” [#560]. The doctor went on to suggest that the MOOC could be further adapted for different audiences, including 1 version for nurses, 1 for community health workers, and 1 for community members. Respondents indicated that having the flexibility to be able to add or remove content to reflect different contextual needs would support a meaningful adaptation that would localize recommendations to better educate their communities. Some respondents suggested that certain content was not relevant for local contexts and could be shortened or was relevant but needed more explanation. For example, a health educator shared:

There is a desire and a need for mental health programming and training, but it was too high level for the community health workers to understand… it wasn't being effective.

#458

Different audiences were also unable to use or access the digital platform due to technological barriers. However, offline and low-tech needs and solutions vary by region and audience. Learners indicated that they were very interested in having the ability to adapt the courses to offline or low-tech options in order to share materials with their communities. In discussing the challenges associated with the technology needs of different audiences, especially community health workers, a doctor in Rwanda stated:

At the Health Center level, probably everyone has access to a laptop or a tablet. For the community health workers it's a little bit hit or miss on if they would have access to a smartphone, especially online if they had to watch it online.

#560

In discussing the need to adapt the course to work specifically for patient communities, a community health worker from the United States said:

I think tools that probably would have been a little bit easier for me to utilize... if we printed material out to hand out to the community... Passing out information is the best way to spread educational resources to individuals… we could have done better with handing out material, reading material for them, and just going over it with them to make sure that they have a better understanding of the pandemic itself.

#615

Learners indicated that having the flexibility and guidance on how to fully adapt courses or parts of courses to different offline and low-tech delivery modalities would help support the spread of the content to a broader range of learners, especially in remote and low-resource settings.

## Discussion

To investigate the potential of MOOCs as a strategy to rapidly educate health professionals across vastly different contexts during public emergencies, we examined how health professionals globally used a newly developed open-source online course series, the COVID-19 Digital Classroom, in their local responses during the first year of the COVID-19 pandemic. Enrollment data showed that health professionals across 104 countries who were engaged in a range of pandemic response activities (including contact tracing, surveillance, testing, treatment, and risk communication and community engagement) sought out and used the online content in their work. This was particularly the case in LMICs where nearly 90% of enrollees were engaged in the COVID-19 response.

In-depth interviews revealed that the COVID-19 Digital Classroom included content areas and features that were useful for learners and institutions across a variety of contexts globally. Interviewees leveraged their networks with other health professionals to share content knowledge from the MOOC to fill gaps in knowledge needed to respond to the unfolding emergency. The interviewees also reported that they had a need for trustworthy health information to help them implement health education training and information initiatives to reach those in their networks more broadly.

Analysis of course enrollment patterns supports the qualitative finding that health professionals shared information within their personal and professional networks, recommending others to enroll in the MOOC. With nearly a third of enrollees reporting that they learned of the course series through an employer recommendation, along with clusters of learners observed at specific organizations, the enrollment data suggest that organizations, including NGOs, government agencies, and health care providers, were using the MOOC to train employees.

The use of the COVID-19 Digital Classroom as an education tool at a range of academic institutions and universities suggests that higher education institutions globally also sought out online course materials about COVID-19 for their health students. These institutions were predominantly located in North America, but included a range of university types, including nursing schools, public health schools, and community colleges.

Despite the intention of the course developers to create an online content platform specifically to support pandemic responses in LMICs, use of the MOOC, as reflected by enrollment analytics, was low relative to that in high- and middle-income countries. A high proportion of enrollees in LMICs learned about the MOOC from direct consortium promotion, suggesting a need for more intentional paths of distribution in lower-resource areas to reach intended audiences. Enrollment patterns also suggest that sharing was less frequent in LMICs; however, we were only able to observe digital enrollment analytics and could not fully track sharing of content through offline paths (eg, printing and sharing of course materials). Our interview findings point to a potential “multiplier effect” of the use of online learning materials in offline contexts beyond that which is tracked through platform analytics [16].

This study was limited in its ability to disentangle demand for the course with the ability to access online content, and was reliant on a small sample size. Nevertheless, the study illuminates the need for more accessible, targeted, and contextualized content to reach communities globally, particularly those in LMICs. This need was recognized by the consortium of course developers, as reflected in the subsequent translation of the course series into additional languages (Arabic, French, Hindi, Portuguese, Spanish, and Swahili). The consortium also sought out collaborations to support the adaptation of content for contextual needs, including through a partnership with the Sierra Leone Ministry of Health and Sanitation to create a toolkit to support the adaptation of the course series for trainers in Sierra Leone.

The findings of this study demonstrate that while online courses are available to health care professionals who are responsible for further disseminating health guidelines within their communities, there is a need for MOOC content that is easier to adapt and share. The health professionals interviewed expressed that they require more support to facilitate the adaptation of the course content for frontline and community health worker training and community education according to their contexts. They wanted the ability to add, remove, and edit components to make the content relevant to their local context and to translate course materials in local languages.

Furthermore, challenges still exist with regard to technology access and digital literacy that limit the potential of open-source online education content, especially in resource-limited contexts [2,12,13]. Our study aligns with prior research that found access problems due to issues of internet connectivity and bandwidth limitations, issues of cellular coverage, literacy gaps, and other administrative challenges [2,12,13,17,18]. In the case of the Digital Classroom course series, different audiences were unable to access the digital platform and share content more broadly due to technological barriers. Making content available for “offline” or low-bandwidth use would help support these learners.

Future work is needed to better understand how the MOOC approach can be delivered and supported in a way that better meets the needs of diverse communities [19]. Efforts should investigate how MOOCs can be better developed for easier modification to meet contextual needs, while likewise examining how content can be used and shared in offline ways and disseminated via alternative modalities of digital delivery to improve access for all. Such investigations are important to ensure that shifts toward online and digital educational approaches that privilege particular languages and paths of distribution that are not available to all do not exacerbate gaps in access to health care and health knowledge globally.

## Data Availability

Some data are available on reasonable request to the corresponding author.

## Abbreviations

LMIC: low- and middle-income country
MOOC: massive open-source online course
NGO: nongovernmental organization
